# Supplementary material for: Isolation, culture, and characterisation of bovine ovarian fetal fibroblasts and gonadal ridge epithelial-like cells and comparison to their adult counterparts
Source: PLoS One. 2022 Jul 8;17(7):e0268467. doi: 10.1371/journal.pone.0268467 (PMC9269465; doi:10.1371/journal.pone.0268467)
Supplement: S1 Fig — After 2 days in culture, sporadic GREL cell clusters (A) and fetal fibroblasts (B) could be observed. GREL clusters grew slowly and formed larger clusters (C) in the subsequent culture days. GREL cells appeared polygonal, and in some areas tightly packed, as previously observed [8]. (D) Fetal fibroblasts grew faster than GREL cells and showed the typical spindle shaped appearance as seen in adult fibroblasts. (E) Alongside wells with pure GREL cells and pure fibroblasts, wells with both cell types could be observed. In these GREL cells formed clearly distinguishable clusters surrounded by fibroblasts. A sterile blunt-ended glass pipette was used to remove the fibroblasts and continue the culture to obtain GREL cells (F). Gestational ages were: (A) 51 and (B-F) 110 days. Bars: A-B, D-F = 100 μm; C = 200 μm. (PDF) [file pone.0268467.s001.pdf]

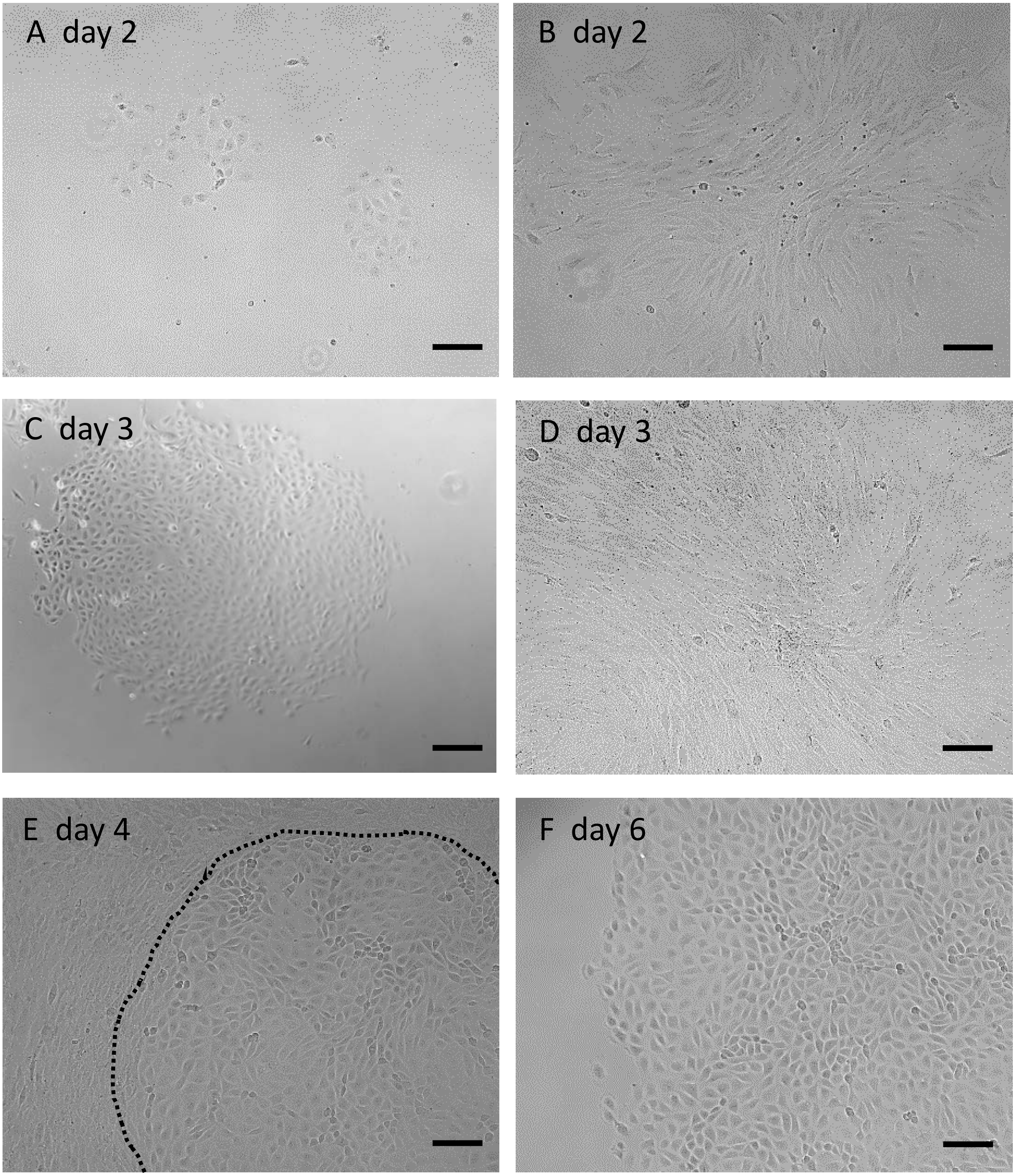


**S1 Fig. Behaviour of bovine fetal ovarian cells cultured on collagen type I coated plates.** After 2 days in culture, sporadic GREL cell clusters (A) and fetal fibroblasts (B) could be observed. GREL clusters grew slowly and formed larger clusters (C) in the subsequent culture days. GREL cells appeared polygonal, and in some areas tightly packed, as previously observed [8]. (D) Fetal fibroblasts grew faster than GREL cells and showed the typical spindle shaped appearance as seen in adult fibroblasts. (E) Alongside wells with pure GREL cells and pure fibroblasts, wells with both cell types could be observed. In these GREL cells formed clearly distinguishable clusters surrounded by fibroblasts. A sterile blunt-ended glass pipette was used to remove the fibroblasts and continue the culture to obtain GREL cells (F). Gestational ages were: (A) 51 and (B-F) 110 days. Bars: A-B, D-F = 100 µm; C = 200 µm.
