# Supplementary material for: Isolation, culture, and characterisation of bovine ovarian fetal fibroblasts and gonadal ridge epithelial-like cells and comparison to their adult counterparts
Source: PLoS One. 2022 Jul 8;17(7):e0268467. doi: 10.1371/journal.pone.0268467 (PMC9269465; doi:10.1371/journal.pone.0268467)
Supplement: S2 Fig — GREL cell clusters were grown on collagen type I coated plates. All 7 GREL cell samples collected for qRT-PCR were from passage 0. Gestational ages were (A) 51, (B) 56, (C) 73, (D, E) 110, (F) 127 and (G) 177 days. Bars: (A-G) = 100 μm. (PDF) [file pone.0268467.s002.pdf]

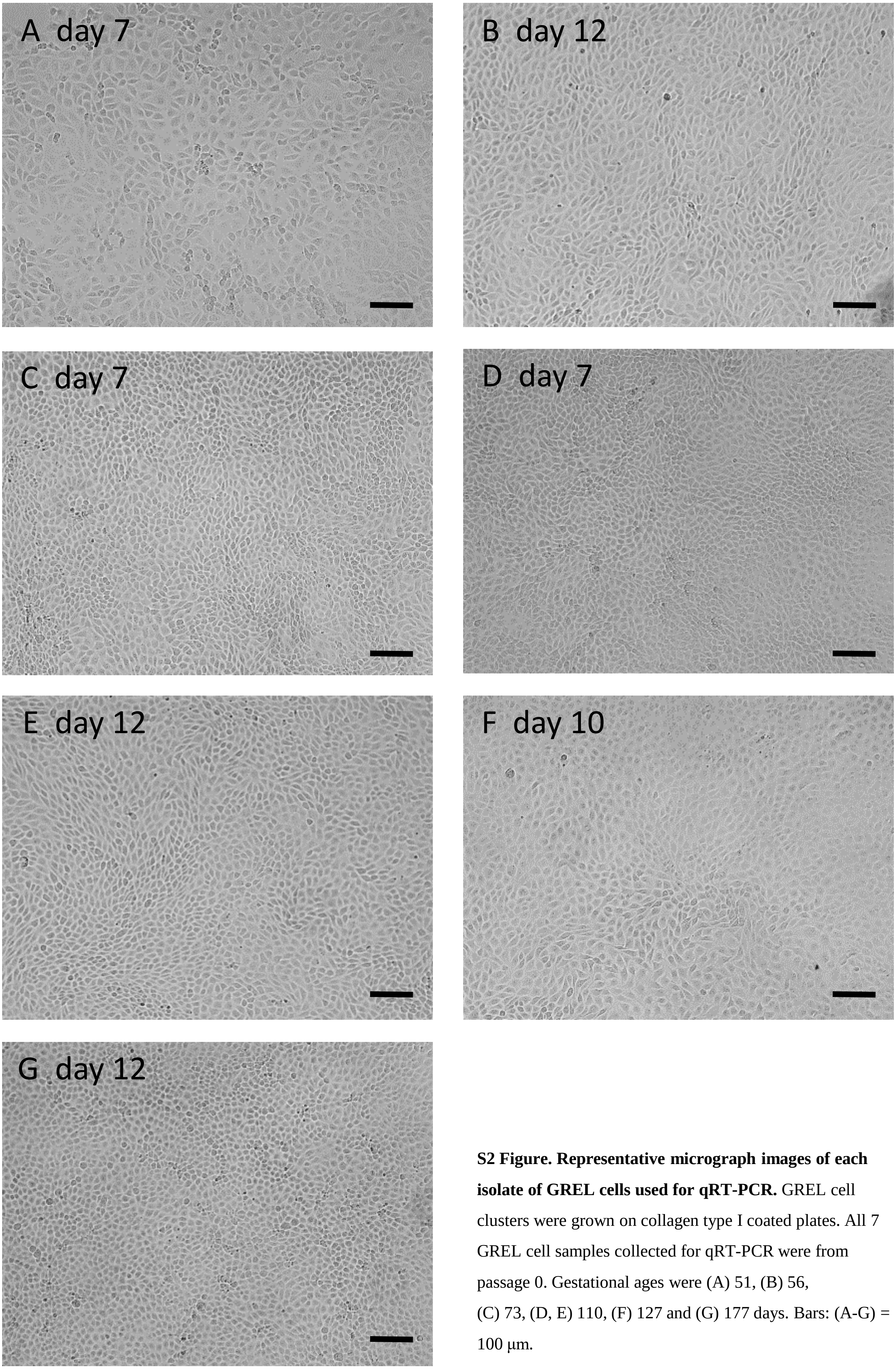

S2 Figure. Representative micrograph images of each isolate of GREL cells used for qRT-PCR. GREL cell clusters were grown on collagen type I coated plates. All 7 GREL cell samples collected for qRT-PCR were from passage 0. Gestational ages were (A) 51, (B) 56, (C) 73, (D, E) 110, (F) 127 and (G) 177 days. Bars: (A-G) = 100 μm.
