# Supplementary material for: Isolation, culture, and characterisation of bovine ovarian fetal fibroblasts and gonadal ridge epithelial-like cells and comparison to their adult counterparts
Source: PLoS One. 2022 Jul 8;17(7):e0268467. doi: 10.1371/journal.pone.0268467 (PMC9269465; doi:10.1371/journal.pone.0268467)
Supplement: S3 Fig — Fetal fibroblasts were grown on collagen type I coated plates and collected on the same day as the corresponding GREL cells for gene expression analysis. All 6 fibroblast samples were from passage 0. Gestational ages were (A) 51, (B) 56, (C) 73, (D, E) 110, (F) 127 and (G) 177 days. Bars: (A-F) = 100 μm. (PDF) [file pone.0268467.s003.pdf]

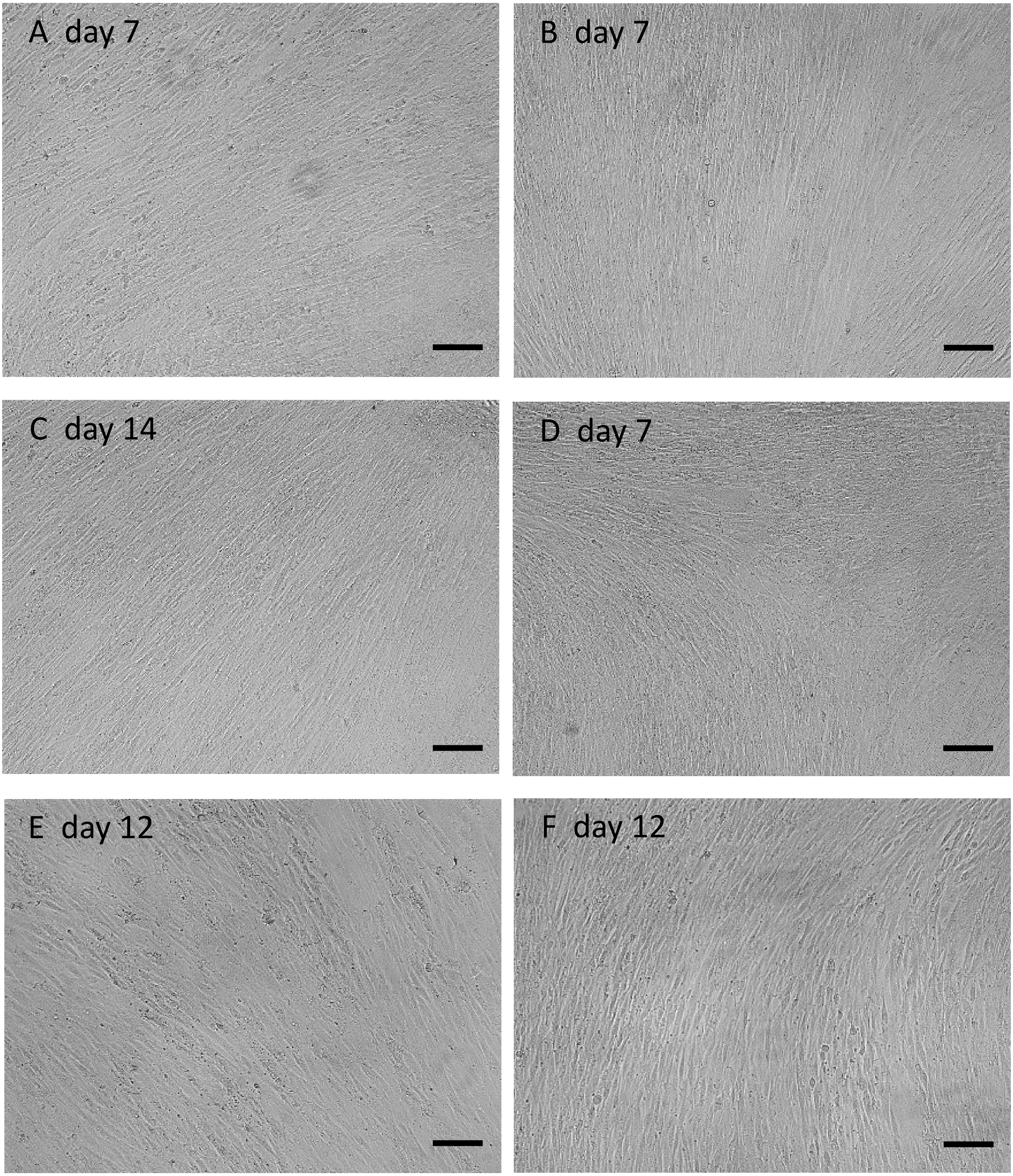

S3 Figure. Representative micrograph images of each isolate of fetal fibroblasts used for qRT-PCR. Fetal fibroblasts were grown on collagen type I coated plates and collected on the same day as the corresponding GREL cells for gene expression analysis. All 6 fibroblast samples were from passage 0. Gestational ages were (A) 51, (B) 56, (C) 73, (D, E) 110, (F) 127 and (G) 177 days. Bars: (A-F) = 100 µm.
