# Supplementary material for: Isolation, culture, and characterisation of bovine ovarian fetal fibroblasts and gonadal ridge epithelial-like cells and comparison to their adult counterparts
Source: PLoS One. 2022 Jul 8;17(7):e0268467. doi: 10.1371/journal.pone.0268467 (PMC9269465; doi:10.1371/journal.pone.0268467)

S4 Fig. Normalised gene expression of genes in GREL cells and fetal fibroblasts. Gene expression levels in cultured GREL (; n = 7 number of samples analysed) and fetal fibroblasts (; n = 6) were normalised to PPIA and RPL32, and they are presented as scatter plots.

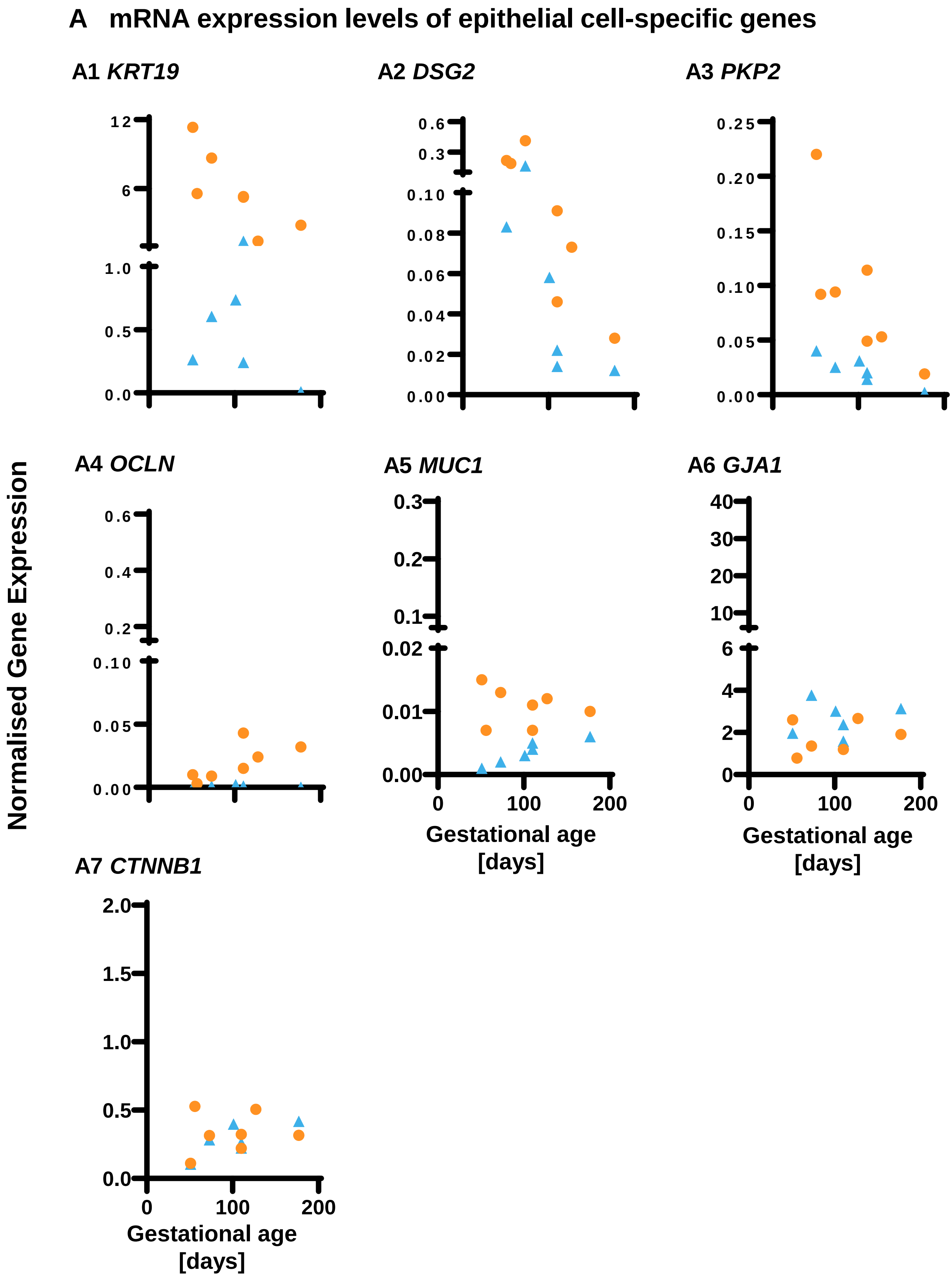
A mRNA expression levels of epithelial cell-specific genes
A1 KRT19
A2 DSG2
A3 PKP2
A4 OCLN
A5 MUC1
A6 GJA1
A7 CTNNB1
Normalised Gene Expression
Gestational age [days]
12
6
1.0
0.5
0.0
0.6
0.3
0.10
0.08
0.06
0.04
0.02
0.00
0.25
0.20
0.15
0.05
0.4
0.2
0.1
0.01
40
30
20
10
4
2
0
100
200
2.0
1.5

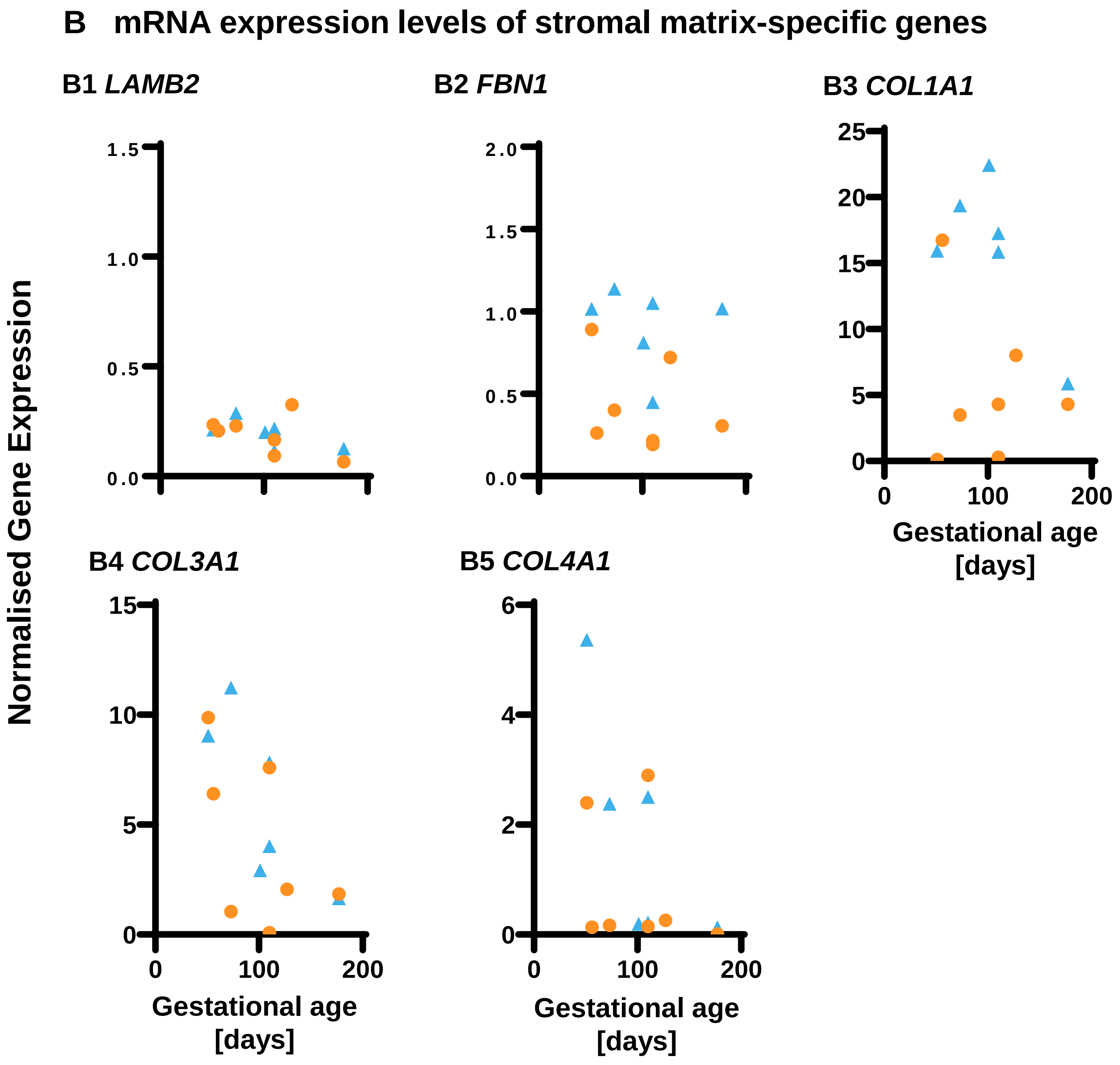
B mRNA expression levels of stromal matrix-specific genes
B1 *LAMB2*
B2 *FBN1*
B3 *COL1A1*
B4 *COL3A1*
B5 *COL4A1*
Normalised Gene Expression
1.5
1.0
0.5
0.0
2.0
25
20
15
10
5
0
6
4
2
100
200
Gestational age [days]

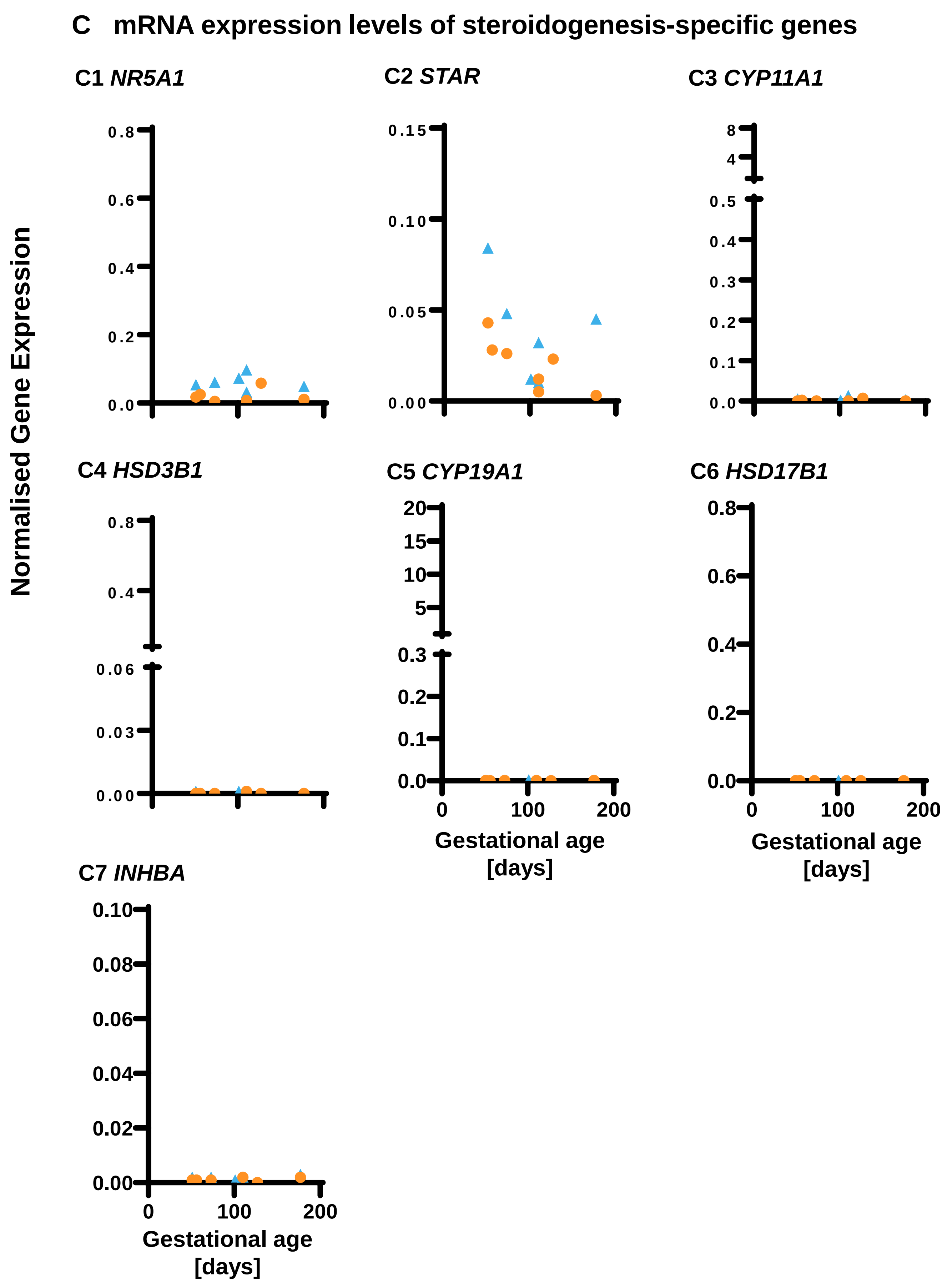
C mRNA expression levels of steroidogenesis-specific genes
C1 NR5A1
C2 STAR
C3 CYP11A1
C4 HSD3B1
C5 CYP19A1
C6 HSD17B1
C7 INHBA
Normalised Gene Expression
Gestational age [days]
0.0
0.2
0.4
0.6
0.8
0.00
0.05
0.10
0.15
0.1
0.3
0.5
4
8
0.03
0.06
5
10
15
20
0.02
0.04
0.08
0
100
200

D mRNA expression levels of receptors in estrogen and TGF-β signalling pathways

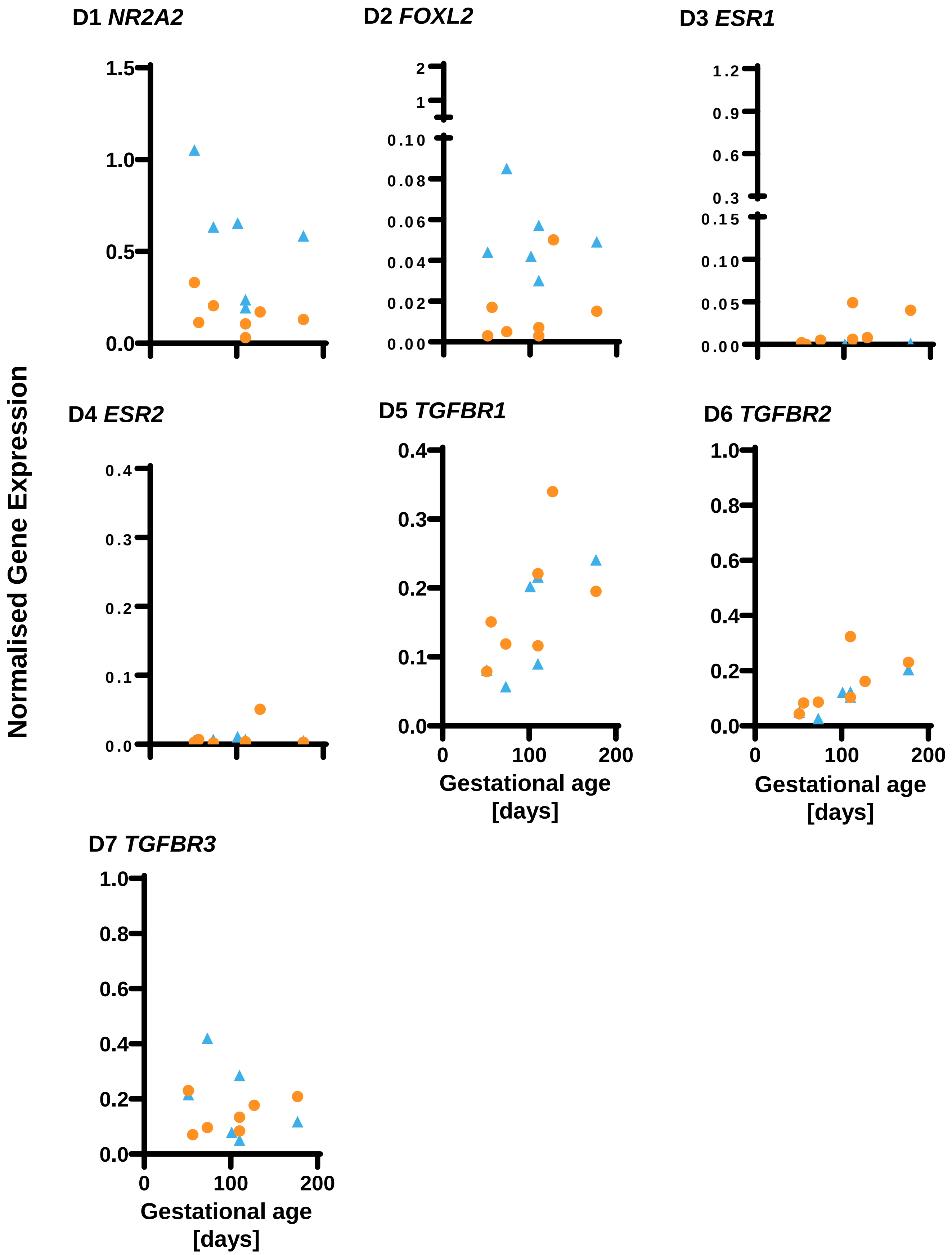

E mRNA expression levels of cell cycle-specific genes

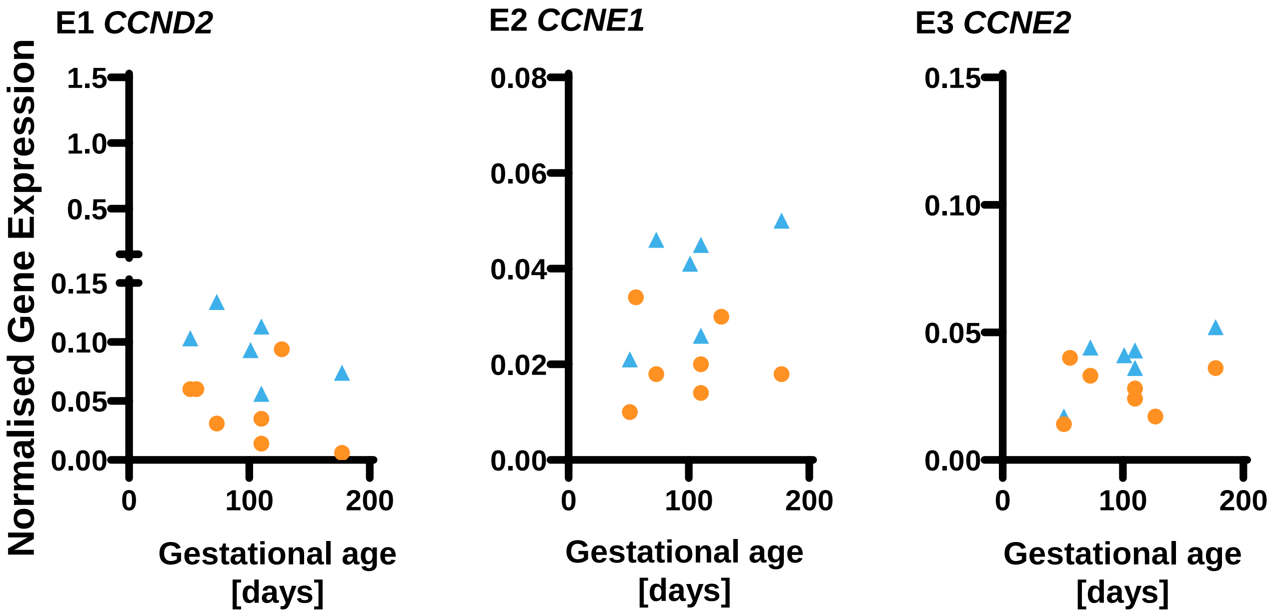

F mRNA expression levels of receptors instem and germ cell-specific genes

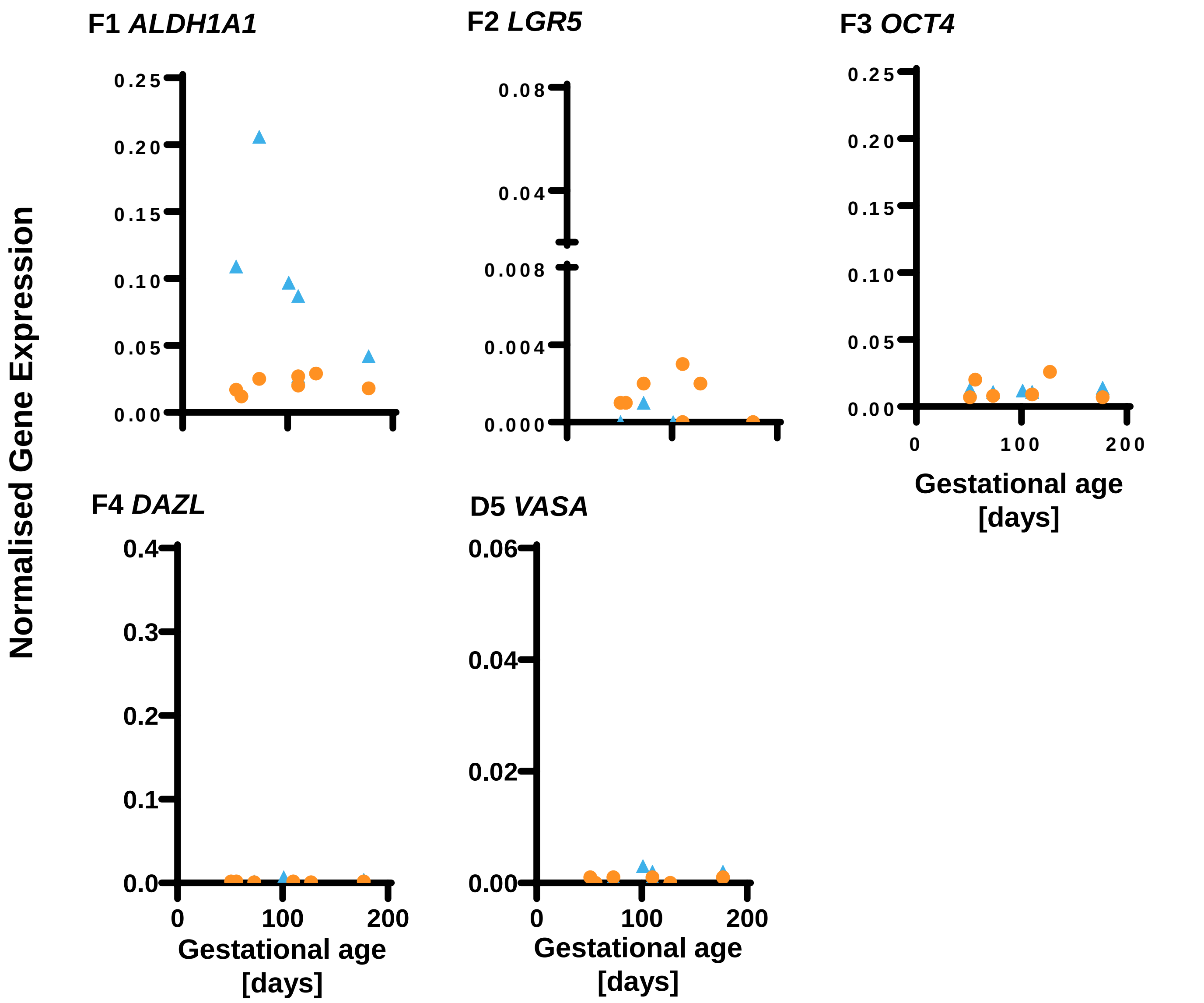

Supplement: S4 Fig — Gene expression levels in cultured GREL (n = 7) and fetal fibroblasts (n = 6) were normalised to PPIA and RPL32, and they are presented as scatter plots. (PDF) [file pone.0268467.s004.pdf]
