# Supplementary material for: Isolation, culture, and characterisation of bovine ovarian fetal fibroblasts and gonadal ridge epithelial-like cells and comparison to their adult counterparts
Source: PLoS One. 2022 Jul 8;17(7):e0268467. doi: 10.1371/journal.pone.0268467 (PMC9269465; doi:10.1371/journal.pone.0268467)

S5 Fig. Normalised gene expression of stromal genes in fetal fibroblasts in the presence of different treatments. Fibroblasts from bovine fetal ovary (n = 5) were cultured with different treatments for 18 h. Fold changes of gene expression to the control groups are presented as mean ± SEM (normalised to *PPIA* and *RPL32*). Significantly different results among groups were determined by one-way ANOVA with Tukey's post-hoc test.

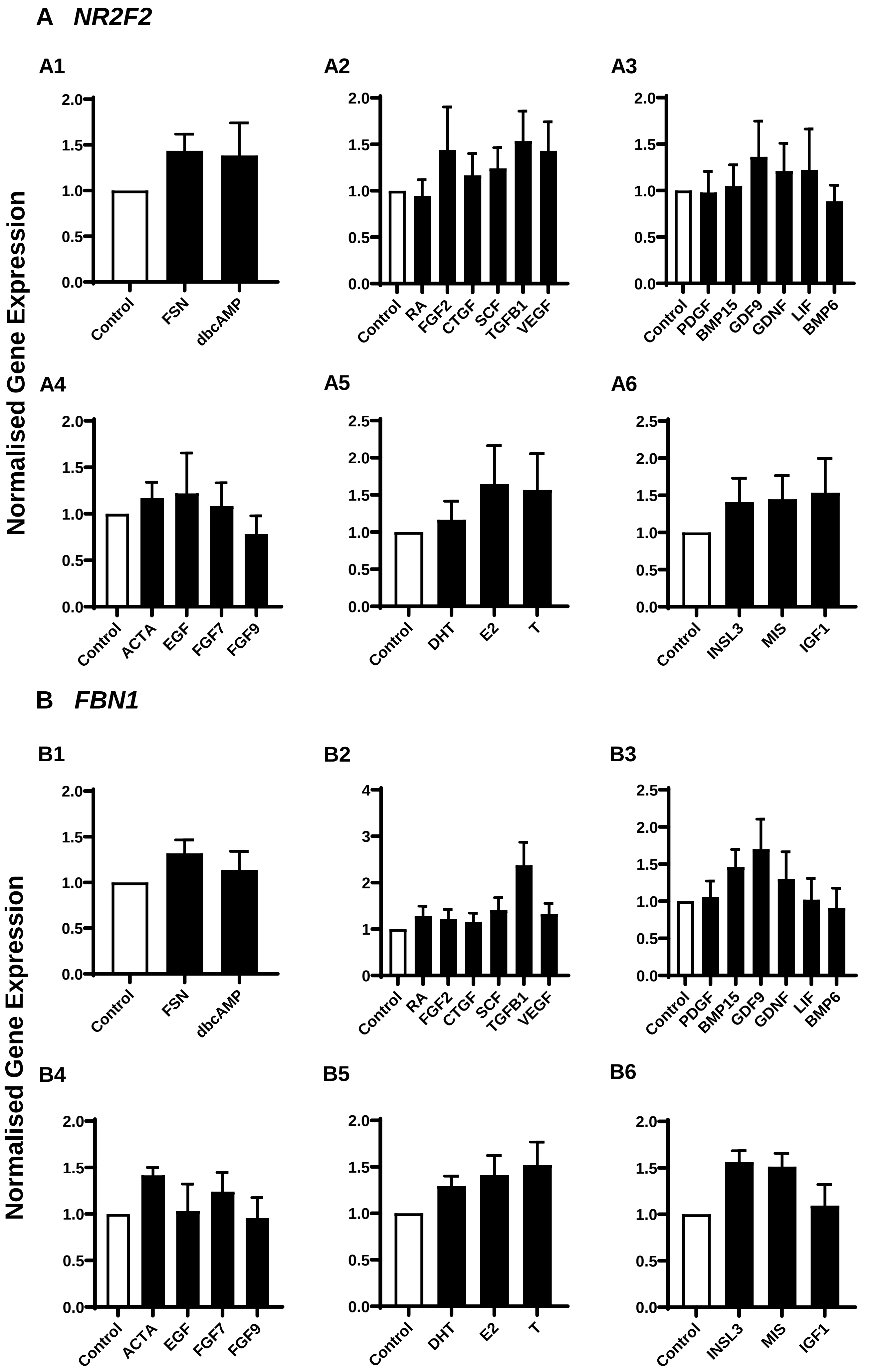
A NR2F2
A1
A2
A3
A4
A5
A6
Normalised Gene Expression
Control
FSN
dbcAMP
RA
FGF2
CTGF
SCF
TGFB1
VEGF
PDGF
BMP15
GDF9
GDNF
LIF
BMP6
ACTA
EGF
FGF7
FGF9
DHT
E2
T
INSL3
MIS
IGF1
B FBN1
B1
B2
B3
B4
B5
B6

C *COL1A1*

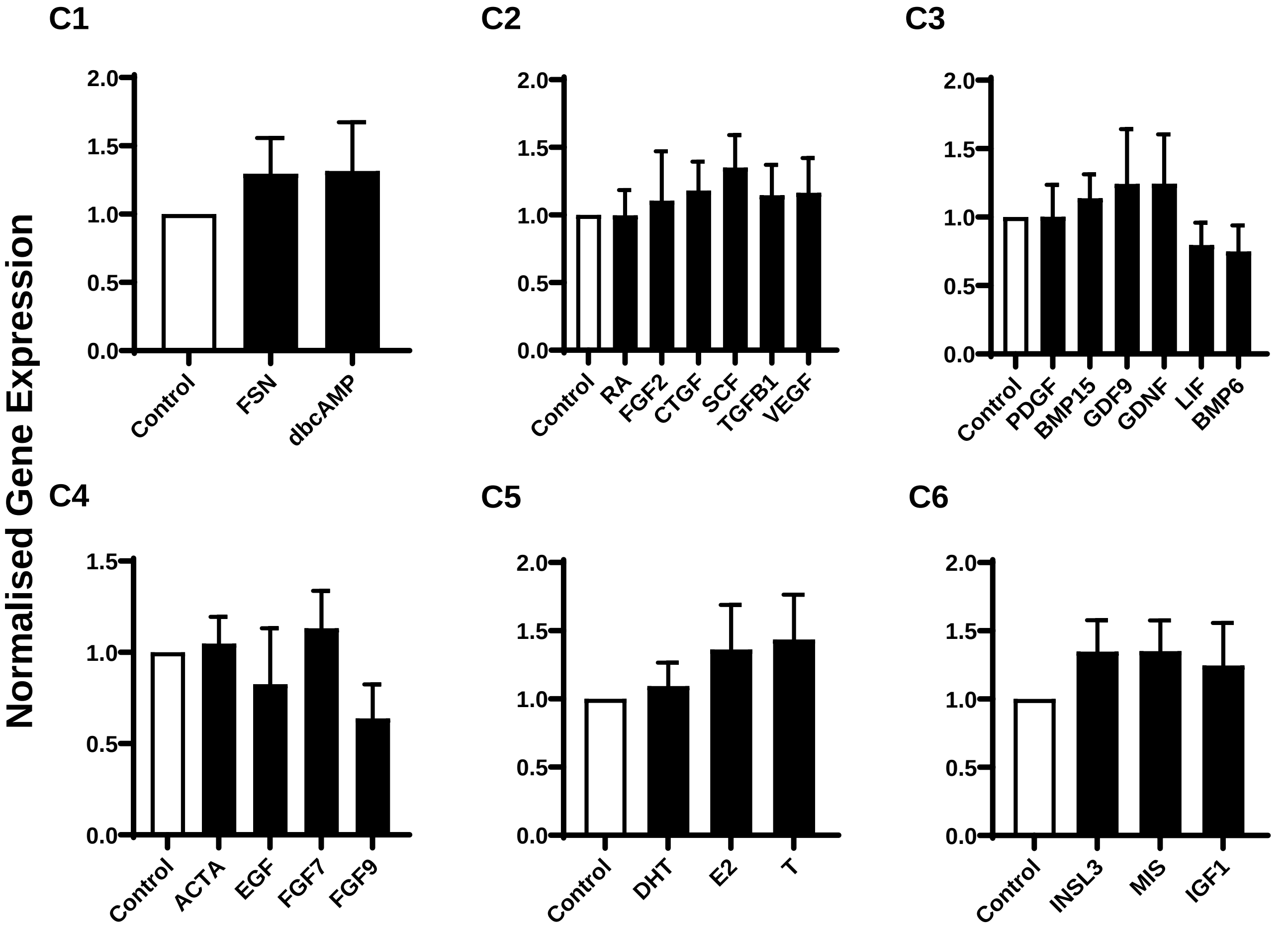

D *COL3A1*

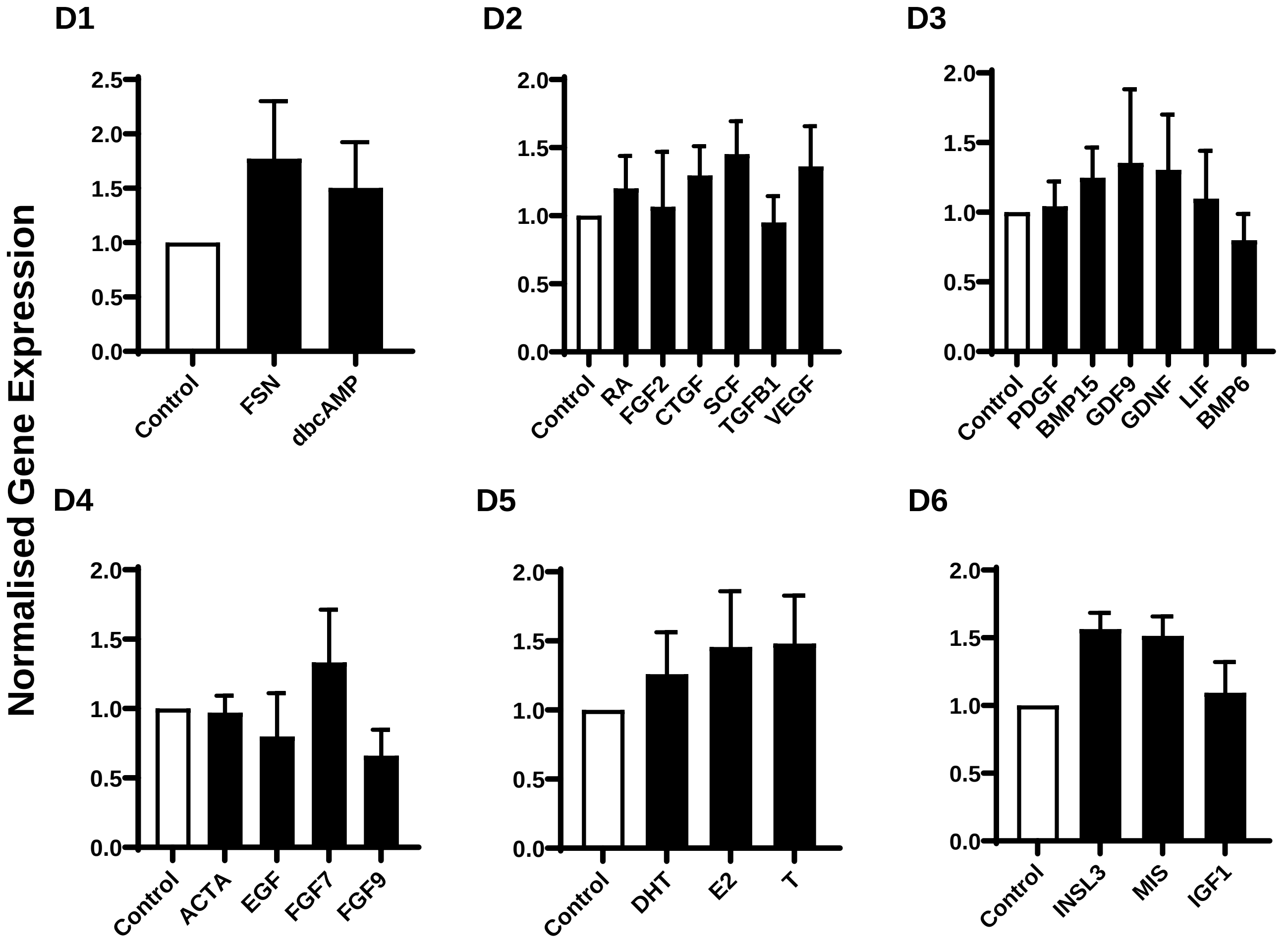

Supplement: S5 Fig — Fibroblasts from bovine fetal ovary (n = 5) were cultured with different treatments for 18 h. Fold changes of gene expression to the control groups are presented as mean ± SEM (normalised to PPIA and RPL32). Significantly different results among groups were determined by one-way ANOVA with Tukey’s post-hoc test. (PDF) [file pone.0268467.s005.pdf]
