# Supplementary material for: Isolation, culture, and characterisation of bovine ovarian fetal fibroblasts and gonadal ridge epithelial-like cells and comparison to their adult counterparts
Source: PLoS One. 2022 Jul 8;17(7):e0268467. doi: 10.1371/journal.pone.0268467 (PMC9269465; doi:10.1371/journal.pone.0268467)
Supplement: S6 Fig — Columns in white colour and black colour show gene expression in fetal fibroblasts cultured in the absence of EGF (n = 11) in a previous study [26] and in the presence of EGF (n = 7) in the current study, respectively. Data of gene expression are presented as mean ± SEM (normalised to PPIA and RPL32). Significantly different results between groups were determined by Student’s t-test. ** P < 0.01. (PDF) [file pone.0268467.s006.pdf]

S6 Fig. mRNA expression levels of stromal genes in fetal fibroblasts cultured in presence or absence of EGF. Columns in white colour and black colour show gene expression in fetal fibroblasts cultured in the absence of EGF (n = 11) in a previous study [Bastian et al, 2016] and in the presence of EGF (n = 7) in the current study, respectively. Data of gene expression are presented as mean ± SEM (normalised to PPIA and RPL32). Significantly different results between groups were determined by Student's t-test. ** $P < 0.01$.

Bastian NA, Bayne RA, Hummitzsch K, Hatzirodos N, Bonner WM, Hartanti MD, et al. Regulation of fibrillins and modulators of TGFbeta in fetal bovine and human ovaries. Reproduction. 2016;152(2):127-37. Epub 2016/05/26. doi: 10.1530/rep-16-0172. PubMed PMID: 27222596

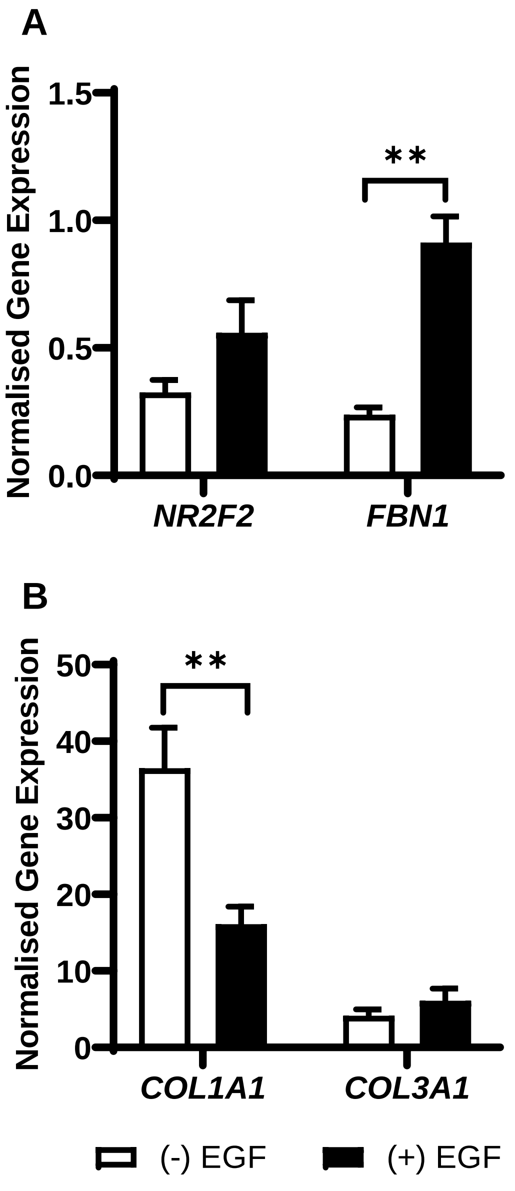
A
Normalised Gene Expression
1.5
1.0
0.5
0.0
**
NR2F2
FBN1
B
Normalised Gene Expression
50
40
30
20
10
0
**
COL1A1
COL3A1
(-) EGF
(+) EGF
