# Supplementary material for: Isolation, culture, and characterisation of bovine ovarian fetal fibroblasts and gonadal ridge epithelial-like cells and comparison to their adult counterparts
Source: PLoS One. 2022 Jul 8;17(7):e0268467. doi: 10.1371/journal.pone.0268467 (PMC9269465; doi:10.1371/journal.pone.0268467)
Supplement: S1 Table — (PDF) [file pone.0268467.s007.pdf]

S1 Table. Treatments of cultured bovine fetal ovarian fibroblasts. Adapted from Bastian et al.

**Treatments**	**Abbreviation**	**Concentration**	**Distributor**
Dihydroxytestosterone	DHT	100 ng/ml	Sigma-Aldrich
Oestradiol	E2	100 ng/ml	Sigma-Aldrich
Testosterone	T	100 ng/ml	Sigma-Aldrich
Insulin-like protein 3	INSL3	100 ng/ml	From Dr. Ross Bathgate – University of Melbourne, Australia
Müllerian inhibiting substance	MIS	10 ng/ml	Biogen Idec Australia Pty Ltd., North Ryde, NSW, Australia
Insulin-like growth factor 1	IGF1	30 ng/ml	GroPep Bioreagents Pty Ltd., Thebarton, SA, Australia
Forskolin	FSK	4.1 µg/ml	Sigma-Aldrich
Dibutyryladenosine cyclic monophosphate	DBcAMP	1 mM	Sigma-Aldrich
Retinoic acid	RA	3 µg/ml	Sigma-Aldrich
Activin A	ACTA	100 ng/ml	R&D Systems
Basic fibroblast growth factor	bFGF	100 ng/ml	Roche Australia Pty Ltd., Thebarton, SA, Australia
Bone morphogenetic protein 6	BMP6	100 ng/ml	R&D Systems
Epidermal growth factor	EGF	10 ng/ml	Boehringer Ingelheim Pty Ltd., North Ryde, NSW, Australia
Fibroblast growth factor 7	FGF7	10 ng/ml	R&D Systems
Fibroblast growth factor 9	FGF9	30 ng/ml	R&D Systems
Platelet-derived growth factor	PDGF	10 ng/ml	R&D Systems
Bone morphogenetic protein 15	BMP15	100 ng/ml	R&D Systems
Connective tissue growth	CTGF	25 ng/ml	Invitrogen/Life Technologies
Stem cell factor	SCF	100 ng/ml	R&D Systems

Transforming growth factor beta 1	TGFβ1	10 ng/ml	R&D Systems
Vascular endothelial growth factor	VEGF	10 ng/ml	R&D Systems
Glial-derived factor 9	GDF9	100 ng/ml	R&D Systems
Glial-cell derived neurotrophic factor	GDNF	100 ng/ml	R&D Systems
Leukaemia inhibitory factor	LIF	10^3 U/ml	Sigma-Aldrich

Bastian NA, Bayne RA, Hummitzsch K, Hatzirodos N, Bonner WM, Hartanti MD, et al. Regulation of fibrillins and modulators of TGFbeta in fetal bovine and human ovaries. Reproduction. 2016;152(2):127-37. Epub 2016/05/26. doi: 10.1530/rep-16-0172. PubMed PMID: 27222596.
