# Supplementary material for: Isolation, culture, and characterisation of bovine ovarian fetal fibroblasts and gonadal ridge epithelial-like cells and comparison to their adult counterparts
Source: PLoS One. 2022 Jul 8;17(7):e0268467. doi: 10.1371/journal.pone.0268467 (PMC9269465; doi:10.1371/journal.pone.0268467)
Supplement: S2 Table — (PDF) [file pone.0268467.s008.pdf]

S2 Table. List of genes and primers used for qRT-PCR.

Gene name	Gene Symbol	Primers (5'→3') (F=forward, R=reverse)	Accession Number	Size (bp)
Ribosomal protein L32	*RPL32*	F : GCCATCAGAATCACCAATCC<br>R : AAATGTGCACACGAGCTGTC	NM_001034783.2	73
Peptidylprolyl isomerase A (cyclophilin A)	*PPIA*	F : CTGGCATCTTGTCCATGGCAAA<br>R : CCACAGTCAGCAATGGTGATCTTC	NM_178320.2	202
Aldehyde dehydrogenase 1 family, member A1	*ALDH1A1*	F : GCGGAAACACAGTGGTTGTC<br>R : GAGAAGAAATGGCTGCCCCT	NM_174239.2	150
Cyclin D2	*CCND2*	F : GGTGGATCTCCTGGCAAAGA<br>R : ACGGTACTGCTGCAGGCTATTC	NM_001076372.1	98
Cyclin E1	*CCNE1*	F : GCCTCAGTATCCTCAGCAAATC<br>R : GAAAATTCTAAGCACCCGACATC	XM_612960.4	84
Cyclin E2	*CCNE2*	F : CCTCATTATTCATTGCTTCCAAAC<br>R : TTCACTGCAAGCACCATCAG	NM_174058.2	129
Collagen type 1 alpha 1	*COL1A1*	F : AAGAAGACATCCCACCAGTCAC<br>R : TAAGTTCGTCGCAGATCACG	NM_001034039.2	149
Collagen type 3 alpha 1	*COL3A1*	F : AGGACACAGAGGCTTTGATGG<br>R : TTTTCCCCCTTTAATCCAGGAG	NM_001076831.1	72
Collagen type 4 alpha 1	*COL4A1*	F : TTTCCAGGCGACTCAGGG<br>R : CGTGCCAATAACAGTTCCGG	XM_580317	106
Catenin Beta 1	*CTNNB1*	F : GAATTGACAAAACTGCTGAATGATG<br>R : GATGGCGTGTCTCGAAGCTT	NM_001076141	102
Cytochrome p450 family 11 subfamily A member 1	*CYP11A1*	F : CACTTTCGCCACATCGAGAA<br>R : TGAATGATATAAACTGACTCCAAATTGC	NM_176644.2	85
Cytochrome p450 family 19 subfamily A member 1	*CYP19A1*	F : GGCTATGTGGACGTGTTGACC<br>R : TGAGAAGGAGAGCTTGCCATG	NM_174305.1	142
Deleted in azoospermia-like	*DAZL*	F : ACGTTTTGCCCAGTGAATGC<br>R : TACCACCGTCTGTATGCTTCTG	NM_001081725.1	98
Desmoglein 2	*DSG2*	F : TGAGACAAAAGCGTGCTTGG<br>R : CAGAGTGTATCTTGGCAATCGG	NM_001192172.2	93
Estrogen receptor 1	*ESR1*	F : GTCCACCTTTTGGAATGTGC<br>R : ATTTTCCCTGGTTCCTGTCC	NM_001001443.1	124

Estrogen receptor 2	*ESR2*	F : TCGACTTCGGAAGTGCTATGAG<br>R : ACCGTTCCTCTTGGTTTTGC	NM_174051.3	136
Fibrillin 1	*FBN1*	F : GGGATGGATTTTGTTCGAGGC<br>R : CATCACTGCAGCTACCTCCATT	NM_174053	126
Forkhead box L2	*FOXL2*	F : AGAATAGCATCCGCCACAAC<br>R : CCCTTCTCGAACATGTCCTC	NM_001031750.1	127
Gap junction alpha-1 protein	*GJA1*	F : TGAGTGCCGTTTACACTTGC<br>R : ACACCAATGACACGACAAGC	NM_174068.2	117
17beta-hydroxysteroid dehydrogenase	*HSD17B1*	F : TGTGGTACTCATTACCGGCTGTT<br>R : CAGCGTGGCATACACTTTGAA	NM_001102365.6	100
3 beta-hydroxysteroid dehydrogenase	*HSD3B1*	F : CCGCGAGAGACCATCATGA<br>R : ACGCTGGCCTGGACACA	NM_174343	68
Inhibin, beta A	*INHBA*	F : ATCATCACGTTCGCGGAATC<br>R : ACTTTGCTCCGGGTCCTGTT	NH_174363.2	144
Keratin 19	*KRT19*	F : CGAGGAGGAAATGAGTGTGCT<br>R : ATCTTGGCTAGGTCGATGCC	NM_001015600.4	90
Laminin subunit beta 2	*LAMB2*	F : CGGGAGTCACACAGAAAGCA<br>R : CCCAGCCCTACTGCATCGT	NM_001046247	64
Leucine rich repeat containing G protein-coupled receptor 5	*LGR5*	F : TTGGGAGATCTGCTTTTCAACA<br>R : TGTGAGGCGCCATTCAAA	NM_001277226	65
Mucin 1	*MUC1*	F : TTCCCAGTGCTTACAGTTGC<br>R : TTGGGCTGCTTTGTGTAGTG	NM_174115.2	100
Nuclear receptor subfamily 2	*NR2F2*	F : ACGGATCTTCCAAGAGCAAG<br>R : TTCCACATGGGCTACATCAG	NM_174402.3	130
Nuclear receptor subfamily 5, group A, member 1	*NR5A1*	F : CAGACCTTCATCTCCATCGTG<br>R : CTTGCCATGCTGAATCTGAC	NM_174403.2	147
Occludin	*OCLN*	F : TTCGACCAATGCTCTCTCAG<br>R : GCGATGCACATCACGATAAC	NM_001082433.2	130
Octamer-binding transcription factor 4	*OCT4*	F : AGGCTTTGCAGCTCAGTTTC<br>R : TTGTTGTCAGCTTCCTCCAC	NM_174580.2	79
Plakophilin 2	*PKP2*	F : AGGAGGTGTGATGGACTGATTG<br>R : TGTCATCTGGCTGGTAATCTGC	NM_001083729.1	76
Steroidogenic acute regulatory protein	*STAR*	F : CAGCAGAAGGGTGTCATCAGA<br>R : GAGAGGACCTGGTTGATGATG	NM_174189.3	152

Transforming growth factor, beta receptor 1	*TGFBR1*	F : CAGAGTGGGAACAAAAAGGTACATG<br>R : CATTGCATAGATGTCAGCACGTT	NM_174621.2	100
Transforming growth factor, beta receptor 2	*TGFBR2*	F : AACGTAGTAACTCCCTGCACTG<br>R : AAACAAACGTGGGTGTGACG	NM_001159566.1	141
Transforming growth factor, beta receptor 3	*TGFBR3*	F : TGCACTTTCCTATCCCACAAGCCG<br>R : CCAGATCATTGAGGCATCCAGCG	XM_005197830.1	197
DEAD (Asp-Glu-Ala-Asp) box polypeptide 4	*VASA*	F : ATGAAGCTGATCGCATGCTG<br>R : TGACGCTGTTCCTTTGATGG	NM_001007819.1	91
