# Supplementary material for: Isolation, culture, and characterisation of bovine ovarian fetal fibroblasts and gonadal ridge epithelial-like cells and comparison to their adult counterparts
Source: PLoS One. 2022 Jul 8;17(7):e0268467. doi: 10.1371/journal.pone.0268467 (PMC9269465; doi:10.1371/journal.pone.0268467)
Supplement: S3 Table — (PDF) [file pone.0268467.s009.pdf]

S3 Table. Pearson correlation coefficients (R) of expression levels of 10 newly examined genes in bovine fetal ovaries and gestational age (n = 27). The intensity of the background color indicates the strength of the significance of the correlation. Blue indicates negative correlations and green indicates positive correlations.

	Ages (days)	*MUC1*	*PKP2*	*CCNE1*	*CCNE2*	*OCLD*	*STAR*	*COL4A1*	*GJA1*	*LAMB2*
MUC1	-0.890[c]									
PKP2	-0.778[c]	0.749[c]								
CCNE1	-0.596[b]	0.531[b]	0.511[b]							
CCNE2	-0.410[a]	0.308	0.351	0.922[c]						
OCLD	-0.047	-0.09	0.086	0.589[b]	0.596[b]					
STAR	0.464[a]	-0.336	-0.217	-0.252	-0.235	-0.368				
COL4A1	0.476[a]	-0.203	-0.013	-0.328	-0.246	-0.141	0.285			
GJA1	0.497[b]	-0.402[a]	-0.251	-0.322	-0.221	0.017	0.236	0.516[b]		
LAMB2	0.594[b]	-0.503[b]	-0.266	-0.239	-0.092	0.219	0.247	0.500[b]	0.462[a]	
HSD17B1	0.667[c]	-0.528[b]	-0.472[a]	-0.391[a]	-0.364	-0.215	0.717[c]	0.473[a]	0.332	0.236

[a] $P < 0.05$, [b] $P < 0.01$, [c] $P < 0.0001$; Pearson correlation tests.
