# Supplementary material for: Isolation, culture, and characterisation of bovine ovarian fetal fibroblasts and gonadal ridge epithelial-like cells and comparison to their adult counterparts
Source: PLoS One. 2022 Jul 8;17(7):e0268467. doi: 10.1371/journal.pone.0268467 (PMC9269465; doi:10.1371/journal.pone.0268467)
Supplement: Attachment — Submitted filename: Reply to reviewer.docx [file pone.0268467.s011.docx]

COMMENT: This manuscript is mainly focused on just technical details of isolation, culture, and gene expression analysis. The authors listed out the differential expression of a list of genes but didn’t explain what it could be the effect of these differential gene expressions make them unique in terms of functionality of the GREL cells. To justify the title of the manuscript, authors must fully characterize the cells by performing an analysis of cell lineage markers, cell doubling time, cell cycle analysis, etc., and compare them with other counterpart cells. This would demonstrate why these cells are morphologically and functionally different from other cells. Since it is just isolation, culture, and gene expression analysis, I feel it could be a short communication or a technical report instead of a full-length research article.

REPLY: It should be appreciated that this is the first time GREL cells have been isolated and cultured and as such this a substantial contribution to field of fetal ovary development. We examined expression of 34 genes and compared those with their counterparts. This is a necessary first step in understanding these cells. Hence at this time there are no lineage markers discovered much less are they available to be monitored.

If you feel it can be a short communication then we think the editor should decide this.

COMMENT 1. There are a few minor typos and grammatical errors throughout. The author must check the grammatical errors thoroughly.

REPLY: Thank you for the suggestion. We have proofed it again.

COMMENT 2. It would give a good look at the gene expression data if it was shown in a heat-map instead of bar charts. Just one glance would give a complete picture of up and down-regulated genes across the various cell types. For example, the heat-map shows down-regulated genes in green and up-regulated genes in red in the heat-map.

REPLY: Yes the heat maps could be useful, but we went to a lot of work to ensure we also had data on expression during the continuum of gestation and those data do not lend themselves readily to a heat map, hence we did not include one.

COMMENT 3. What is the reason for adding 3 ng/ml EGF while isolating GREL and fibroblast cells?

REPLY: We found that GREL cells, being an epithelial cell, needed EGF to maintain the morphological phenotype.

COMMENT 4. Why is the incubation temperature at 38.5 c? is there any specific reason to keep them at that temperature?

REPLY: The bovine body temperature is 38.5 C.

COMMENT 5. How pure these cells are? how did the authors make sure that they didn’t pick up other cells while scrapping off the colonies? since PCR is a very sensitive technique, authors may get the expression of the genes from the cross-contaminated cells as well. Did the authors include fibroblast lineage marker genes as a control to check the purity of the GREL cells in the PCR panel?

REPLY: Yes its possible that any contamination could lead to a PCR signal. However, we were using quantitative PCR and not using it for detection. So the comment about the sensitivity of PCR is not so relevant in this situation. Also Figs S2 and S3 show photos of each preparation of cells that were actually used in these experiments and clearly they are highly enriched for their respective cell types.

In any event we said ‘In this study *NR2F2*, *FBN1*, *COL1A1* and *COL1A3* were used as the marker genes for the stromal fibroblasts in the ovary’ and these were higher in fibroblasts than GREL cells. We are confident of our results.

COMMENT 6. Did the authors check the genomic DNA contamination by including non-RT controls? If yes it must be included in the methods section.

REPLY: Yes. We assumed this was a routine control that everyone did. In any event as we reported we used primers spanning introns. We now include this information in the methods (line 207).

COMMENT 7. It is interesting to see the effect of TGF-b, and EGF treatment on gene expression but the authors didn’t explain the functional outcome. The manuscript lacks explanations for functional aspects although they are significant findings.

REPLY: We said ‘TGFβ1 and EGF may remodel ECM by regulating gene expression in fetal fibroblasts.’ Given that we do not know about the in vivo expression of these molecules we feel in premature to speculate too much at this stage about the meaning of the results for the in vivo situation.

COMMENT 8. X and Y axis titles must be present for every graph.

REPLY: We prefer to have the X-axis labelled for the Figs as a whole not as individual parts of the Fig.

9. In the figure, S4 scatter plot labels are missing, what are the blue and orange dots represent? What does n mean? the number of colonies or number of samples? please mention it in the figure legend.

REPLY: These are now clarified in the legend.
